# Another Look at Succimer: Cognitive Deficits May Be Reversible After All

**Published:** 2007-02

**Authors:** Cynthia Washam

Clinicians for years have used chelation to treat lead poisoning without knowing whether it prevented cognitive impairment in lead-exposed children. A recent study of chelation therapy now brings new hope to parents of children exposed to lead **[*EHP* 115:201–209; Stangle et al.]**. The Cornell University study is thought to be the first to show that chelation can alleviate cognitive deficits caused by lead exposure. That finding contradicts the most comprehensive chelation study to date, in which scientists at the NIEHS found no cognitive benefits of the therapy.

Chelation’s known effect is to cause lead and other metals to be removed quickly from the blood and excreted in urine and feces. The treatment originally was used to prevent death from toxic exposures. Today, though, with the phaseout of leaded gasoline, solders, and paint, nonoccupational exposures are at much lower levels, and typically come from lead-bearing paint and dust in old houses.

In young children, however, even low lead levels can cause learning disabilities, attention difficulties, and antisocial behavior. Clinicians use chelation in children to minimize that risk, despite uncertainties about its effects in this regard. Treatment is recommended by the CDC if the child’s blood lead level exceeds 45 μg/dL. Yet a CDC survey showed many children are treated for levels as low as 10 μg/dL.

The Cornell researchers tested the commonly used chelation drug succimer on juvenile rats fed lead doses that simulated moderate and high childhood exposures. For the lead-exposed rats, chelation was linked with an effective lessening of problems in cognition and emotionality, with a more complete normalization of behavior seen in the moderately exposed rats. An unexpected finding was that rats not exposed to lead but treated with succimer showed cognitive deficits similar to those of untreated rats with high lead levels during early development.

The authors believe that succimer might similarly improve cognition in lead-exposed children if a regimen could be identified that sufficiently reduces brain lead. Succimer’s reduction of brain lead lags behind its effect on blood lead. The authors suggest that the failure of the NIEHS study to show any cognitive benefits of succimer may reflect the small reduction in blood lead—and even smaller reduction in brain lead—achieved by the treatment relative to placebo.

The Cornell team could not explain why succimer produced lasting adverse effects in rats not exposed to lead, but speculated it might be related to the drug’s effect on essential metals such as iron and zinc, which are necessary for proper brain development. Their finding led them to warn against using chelation in children who do not have elevated tissue levels of lead or other heavy metals.

## Figures and Tables

**Figure:**
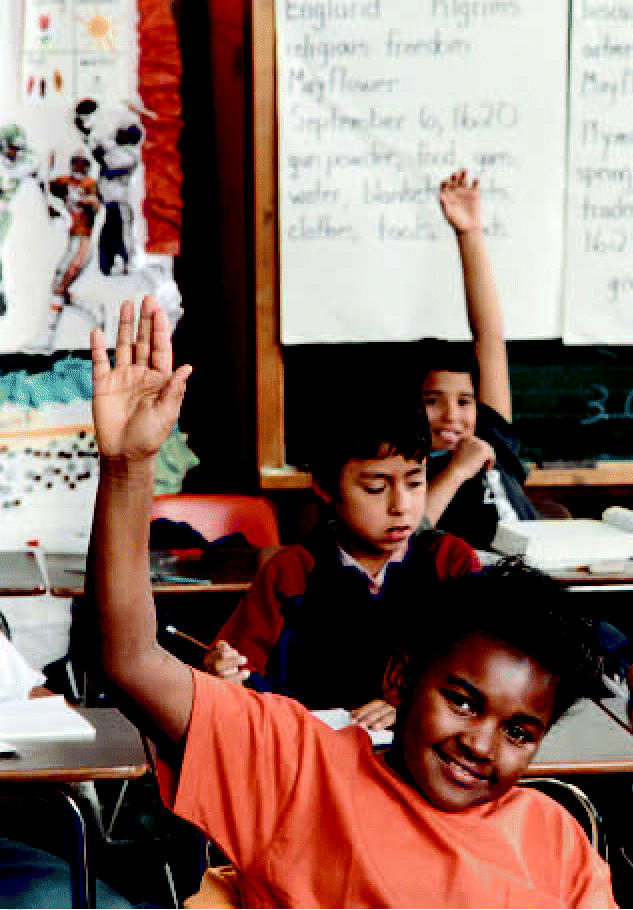
Succimer for success? A new rat study suggests that chelation may negate some cognitive effects of lead exposure.

